# Inherently Antimicrobial P(MMA-*ran*-DMAEMA) Copolymers Sensitive to Photodynamic Therapy: A Double Bactericidal Effect for Active Wound Dressing

**DOI:** 10.3390/ijms24054340

**Published:** 2023-02-22

**Authors:** Orlando Santoro, Miryam Chiara Malacarne, Francesco Sarcone, Luca Scapinello, Stefania Pragliola, Enrico Caruso, Viviana Teresa Orlandi, Lorella Izzo

**Affiliations:** 1 Dipartimento di Biotecnologie e Scienze della Vita, Università degli Studi dell’Insubria, Via J.H. Dunant, 3, 21100 Varese, VA, Italy; 2 Dipartimento di Chimica e Biologia, Università degli Studi di Salerno, Via Giovanni Paolo II, 128, 85085 Fisciano, SA, Italy

**Keywords:** antimicrobial materials, photosensitizers, BODIPY, antimicrobial photodynamic therapy (aPDT), photoinactivation

## Abstract

In this work, two compounds belonging to the BODIPY family, and previously investigated for their photosensitizing properties, have been bound to the amino-pendant groups of three random copolymers, with different amounts of methyl methacrylate (MMA) and 2-(dimethylamino)ethyl methacrylate (DMAEMA) in the backbone. The P(MMA-*ran*-DMAEMA) copolymers have inherently bactericidal activity, due to the amino groups of DMAEMA and to the quaternized nitrogens bounded to BODIPY. Systems consisting of filter paper discs coated with copolymers conjugated to BODIPY were tested on two model microorganisms, *Escherichia coli* (*E. coli*) and *Staphylococcus aureus* (*S. aureus*). On solid medium, irradiation with green light induced an antimicrobial effect, visible as a clear inhibition area around the coated disks. The system based on the copolymer with 43% DMAEMA and circa 0.70 wt/wt% of BODIPY was the most efficient in both bacterial species, and a selectivity for the Gram-positive model was observed, independently of the conjugated BODIPY. A residual antimicrobial activity was also observed after dark incubation, attributed to the inherently bactericidal properties of copolymers.

## 1. Introduction

Skin and soft tissue represent sites commonly colonized by opportunistic pathogens, often responsible for infections in pressure ulcers, burst, or burn wounds [1]. In the initial infection stage, Gram-positive bacteria, which form the majority of skin microbiota (e.g., *Staphylococcus aureus*, *Staphylococcus epidermidis*), are generally predominant, while Gram-negative ones (e.g., *Escherichia coli*, *Pseudomonas aeruginosa*) can be involved in the later stages and worsen tissue damage [2]. To prevent the colonization of bacterial wounds, different wound dressings have been developed, with both natural and synthetic materials in the form of sponges, hydrogels hydrocolloids, films, or membranes and, as might be expected, each of these approaches has advantages and disadvantages [3]. Among these materials, films are interesting because they are difficult for bacteria to colonize, allow monitoring of healing, and are painless [4].

We have previously demonstrated that copolymers made of a block of monomethoxypolyethylene glycol (mPEG) linked to methyl methacrylate’s (MMA) and 2-(dialkylamino)ethyl methacrylate’s (DAAEMA) copolymer chains [mPEG-P(MMA-ran-DAAEMA)*_n_* (*n* = 1, 2, 4)], can be easily obtained via atom transfer radical polymerization (ATRP) and ARGET-ATRP [5,6].

Such copolymers were bactericidal against both Gram-positive and Gram-negative bacteria, and their activity was correlated to the copolymer structure, as well as to the chemical composition. A deep investigation evidenced three fundamental aspects relative to their mechanism of action: (i) the autoionization of the polymer film surfaces when in contact with bacteria [7,8,9,10,11,12]; (ii) the capability of ionized films to remove Ca^2+^ and Mg^2+^ from the outer membrane (OM) of Gram-negative bacteria, increasing its permeability [13]; (iii) the overexpression of some key genes of the synthesis and maintenance of the OM, as well as regulators of cellular response to oxidative stress [14]. Although the bactericidal mechanism killed 99.98% ± 0.01 of the total population of *E. coli*, the latter finding explains the experimental evidence that films immersed in a suspension of bacteria reach higher activity after 5 h, with the attempt of microorganisms to limit the damage to the OM during this time. In this respect, a desirable implementation of the system would be a chemical modification, that allowed an immediate, strong bactericidal action, coupled to the long-term one already present.

Generally, antimicrobial properties of films can be improved by incorporating antimicrobial agents, nanoparticles, or natural products [15]. However, to prevent the selection of antibiotic-resistant pathogens and their environmental spreading, reduced/avoidance of the use of antibiotics is highly desirable.

To this end, among the new antimicrobial techniques, light-based approaches are receiving much attention. In particular, antimicrobial photodynamic therapy (aPDT) combines the use of photosensitizers (PS) with visible light in the presence of oxygen, to cause oxidative stress responsible for killing microorganisms. After irradiation with light at a specific wavelength, the PS passes from the ground state (S0) to the excited state (S1). In this state, through intersystem crossing (ISC) it can undergo a spin inversion, reaching the excited triplet state (T1). From T1, PS is able to transfer energy to molecular oxygen through two processes, called Type I and Type II. In Type I reactions the PS interacts directly with a substrate [16], such as the cell membrane or a molecule, thus transferring a proton or an electron to the substrate to form radicals which will then react with O_2_ to form the superoxide anion (O_2_^•-^), the hydroxyl radical (OH^•^), and hydrogen peroxide (H_2_O_2_) [17]. Alternatively, PS can interact with molecular oxygen (O_2_), thus leading to the formation of singlet oxygen (^1^O_2_) in what is called a Type II reaction. In both cases the oxidant species interact with microbial macromolecules, including proteins, lipids, and nucleic acids, and compromise cell structures, leading to cell death [18].

A further issue that makes the management of skin infections more difficult is the formation of microbial biofilms on tissues and/or clinical devices. Biofilms are well-organised communities where microbial cells communicate with each other to establish a three-dimensional structure able to protect and preserve the entire microbial population. Since, biofilms favour the increase in tolerance of microorganisms to antimicrobial treatments, the photodynamic approach could prevent biofilm formation and the consequent chronic infections [19].

Among different families of antimicrobial PSs, 4,4-difluoro-4-bora-3a,4a-diaza-s-indacenes and boron-dipyrromethenes (BODIPYs) have been recently considered. They can be used in aPDT after appropriate modifications [20], for instance introducing a heavy atom (i.e., iodine or bromine) at the *β* position of the pyrrole moieties, that highly decreases BODIPY fluorescence, with a corresponding increase in the production of ^1^O_2_.

Interestingly, PSs can be linked to amino groups, and the latter, acquiring a positive charge, display better interactions with the OM of microorganisms [21], working as biocidal groups themselves.

Considering the long-term bactericidal activity of copolymers containing amino-pendant groups, as mentioned above, and the antimicrobial activity of PSs once activated by light, in this work, two BODIPYs, A and B, have been bound to the amino-pendant groups of DMAEMA units in P(MMA-*ran*-DMAEMA) copolymers, having different amounts of MMA and DMAEMA. The two BODIPYs have been previously tested against *S. aureus*, *P. aeruginosa*, and *Candida albicans* [22].

Antimicrobial systems, consisting of filter paper discs coated with copolymers conjugated to BODIPY, were tested on *S. aureus* and *E. coli* to evaluate the combination of antimicrobial activity “on demand” by irradiation of PSs, combined with the intrinsic antimicrobial activity of the copolymer.

## 2. Results and Discussion

### 2.1. Synthesis of Copolymer Conjugated with Photosensitizers

Linear copolymers of methyl methacrylate (MMA) and DMAEMA (P(MMA-*ran*-DMAEMA)), exhibiting different molar fractions of the bactericidal monomer DMAEMA, were synthesized by activators regenerated by electron transfer (ARGET-ATRP) [23,24,25,26], using CuBr_2_/2,2′-bipyridyl (Bipy) as a catalyst system. As this polymerization technique allows for the use of copper at ppm levels, the purification of the resulting polymers to remove catalyst residues is not necessary. The reactions were carried out at 60 °C in toluene, in the presence of Sn(II) octoate (Sn(Oct)_2_) and α-bromoisobutyryl bromide (BiBB) as the reducing agent and the radical initiator, respectively (Table 1). Such reaction conditions were selected to favor the formation of random copolymers, with compositions reflecting those of the monomers in the feed. In fact, materials displaying regions with high DMAEMA concentrations (gradient copolymers) were not desired, since this would disfavor the biding of the BODIPY, due to steric hindrance. The composition of the copolymers was found to be in the range of 29 to 51 mol%, as determined by ^1^H NMR spectroscopy (Figures S1–S4). Albeit the intrinsic polymer antimicrobial activity only slightly increases for DMAEMA fractions above 20 mol% [5], higher concentrations of such comonomer would provide binding sites for the PS, as well as maintaining a certain number of pendant free dimethylamino groups.

Due to the higher rigidity of the backbone, the copolymer glass transition temperature (T_g_) increased with the molar fraction of MMA (i.e., from 72 to 90 °C for **CoPol1** and **CoPol3**, respectively—Figures S5–S7). Nevertheless, all values were lower than 100 °C, suggesting a certain chain flexibility, thus indicating such copolymers to be suitable materials to produce films.

**Scheme 1:**
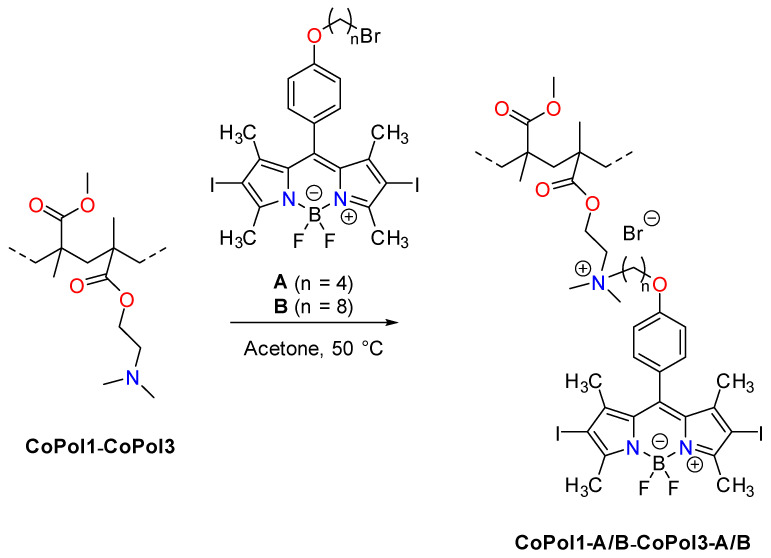
Conjugation of P(MMA-*ran*-DMAEMA) copolymers with the BODIPYs, **A** or **B**. [4,4-difluoro-2,6-diiodio-1,3,5,7-tetramethyl-8-(4-(4-bromobutoxy) phenyl)4-bora-3a,4a-diaza-s-indacene (**A**); 4,4-difluoro-2,6-diiodo-1,3,5,7-tetramethyl-8-(4-[(8-bromooctoxy] phenyl)4-bora-3a,4a-diaza-s-indacene (**B**)].

The BODIPYs chosen for this study are reported in Scheme 1 [27]. Due to their neutral character, as reported in the literature for different PS families, the chosen BODIPYs should be optimal for selectively inactivating Gram-positive bacteria [28]. In addition, the linking of such compounds with polymers with intrinsic antimicrobial activity could widen the spectrum of microbial targets to Gram-negative bacteria, and/or increase the killing rate induced by their photoactivity on Gram-positive bacteria.

The long alkyl chains (4 and 8 carbon atoms for **A** and **B**, respectively) introduced in the structure have been selected to act as spacers between the polymer and the bulky PS, hence favoring the conjugation of the latter with the DMAEMA amino groups along the chain, despite the steric hindrance of the condensate BODIPY rings.

**Figure 1:**
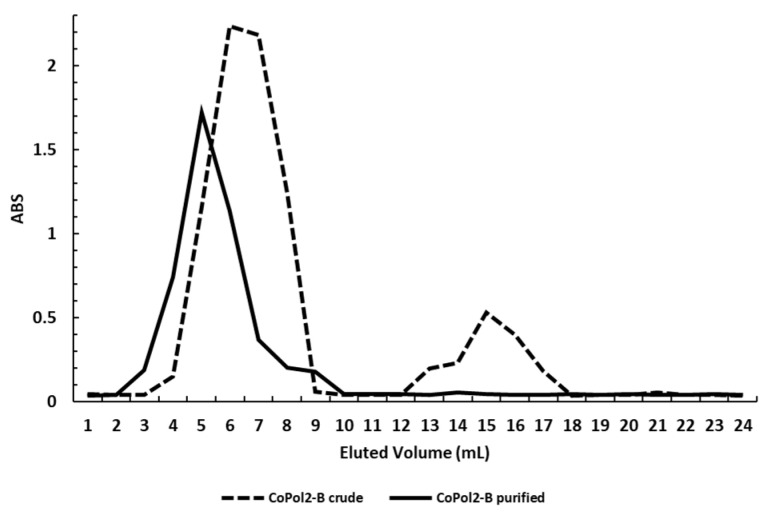
Elution of the copolymer P(MMA-*ran*-DMAEMA) linked to BODIPY (Table 2, entry 4), through a Sephadex LH 20 column, eluted with methanol. The first peak corresponds to the conjugate copolymer, the second fraction to the free BODIPY.

The copolymers were conjugated with **A** and **B** via nucleophilic substitution in acetone. The reaction led to the quaternization of the dimethyl-amino pendant groups of the DMAEMA units. To completely remove unbound BODIPY, the crude copolymers were washed several times with diethyl ether and passed through a *Sephadex^®^ LH-20* column (see experimental part). In Figure 1, spectrophotometric data of each fraction collected are shown before and after purification, to assess that all the unreacted BODIPY was completely removed by the purification processes.

The content of PS in each copolymer was evaluated spectrophotometrically (Table 2). Interestingly, the amount of PS decreased with the molar fraction of DMAEMA incorporated into the parental material. This is probably due to the high steric encumbrance of the BODIPYs, hampering their reaction with amino groups. Furthermore, the length of the BODIPY side-chain had only a modest influence on its conjugation efficiency [29]. Notably, the glass transition temperatures of these materials were found to be higher than those of their parental copolymers (Figures S8–S13). This was attributed to an increased rigidity of the polymer chains, due to the conjugation with the PS, and the proposed formation of inter-chain interactions due to the zwitterionic BODIPY molecules.

### 2.2. Antimicrobial Activity

**Figure 2:**
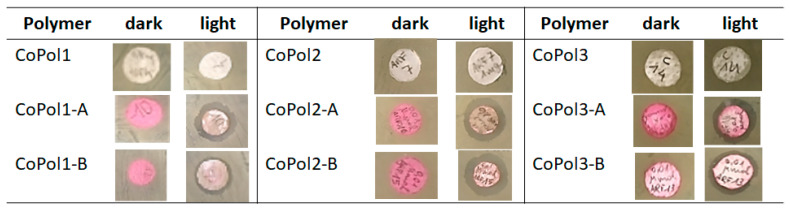
Representative images of antimicrobial assay on *E. coli* K12-MG1655. Bacterial cells were inoculated on LB agar and paper disks were placed on solid medium. Disks were prepared with polymers (**CoPol1**, **CoPol2**, **CoPol3**) or polymers with BODIPYs A and B, respectively. After 2 h incubation at 37 °C, petri dishes were dark incubated or irradiated under green light. After overnight incubation at 37 °C, the antimicrobial activity was visible as a clear inhibition growth halo. The experiments were performed three times with independent bacterial cultures.

We studied the antimicrobial effects of P(MMA-*ran*-DMAEMA) copolymers conjugated with BODYPYs against two model microorganisms: *Escherichia coli* K12-MG1655 and *Staphylococcus aureus* ATCC 6538P, as representatives of Gram-negative and Gram-positive bacteria, respectively. In conventional aPDT, the antibacterial effect(s) of PS is achieved through the development of toxic reactive oxygen species (ROS), which arise from molecule excitation by light of suitable wavelength in aerobic environments. aPDT could thus be considered an antimicrobial tool “on demand”, as the effect is conditioned by the simultaneous presence of the three partners (PS, light, and oxygen). Paper discs with a diameter of 10 mm were coated with the suitable copolymer solutions by a spin coater device, to reach a final concentration of BODIPYs of 0.01 μmol. An example of an experimental outcome in *E. coli*, after overnight incubation at 37 °C, is shown in Figure 2. If no inhibition growth was observed in the absence of light irradiation (Figure 2, A), a clear halo was visible around the paper disc coated with copolymer–BODIPY in the irradiated sample (Figure 2, B).

The different copolymers showed similar antimicrobial activity, irrespective of copolymer and BODIPY (Table 3).

**Figure 3:**
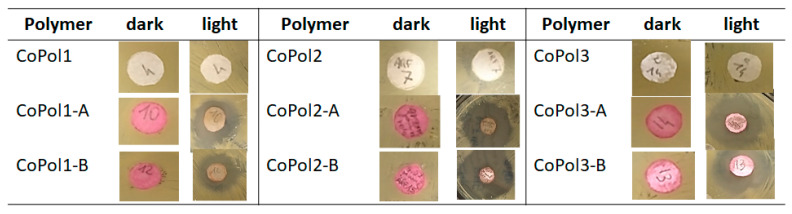
Representative images of antimicrobial assay on *S. aureus* ATCC 6538P. Bacterial cells were inoculated on LB agar and paper disks were placed on solid medium. Disks were prepared with polymers (**CoPol1**, **CoPol2**, **CoPol3**) or polymers with BODIPYs A and B, respectively. After 2 h incubation at 37 °C, petri dishes were dark incubated or irradiated under green light. After overnight incubation at 37 °C, the antimicrobial activity was visible as a clear inhibition growth halo. The experiments were performed three times with independent bacterial cultures.

As shown in Figure 3 and Table 4, *S*. *aureus* appeared more sensitive than *E. coli* to photoinactivation by the copolymer-based materials after irradiation under green light. With all the materials, the diameters of the halo inhibitory zones were greater than that observed in the Gram-negative model.

Furthermore, no bacterial killing effect was observed in the dark incubated controls. In addition, the combination of the **CoPol2** copolymer, with the BODIPY characterized by the eight-carbon alkyl chain (**B**), showed the highest antimicrobial activity. It is worth noting that the amount (μmol) of PS employed was kept constant for each polymer sample, so that a different mass of the parental copolymer was used for the preparation of the disks. Thus, the higher antimicrobial effect exhibited by **CoPol2-B** could be due to the morphology of the coating (i.e., a higher number of PS moieties exposed on the surface of the sample) deriving also from a different polymer conformation in solution when preparing the coated disk [5,30]. Further experiments are needed to verify if the mentioned effects are strain dependent, suggesting a selective activity against Gram-positive bacteria.

The inhibition of bacterial growth close to the disks suggest a possible diffusion of reactive oxygen species or of PSs.

**Figure 4:**
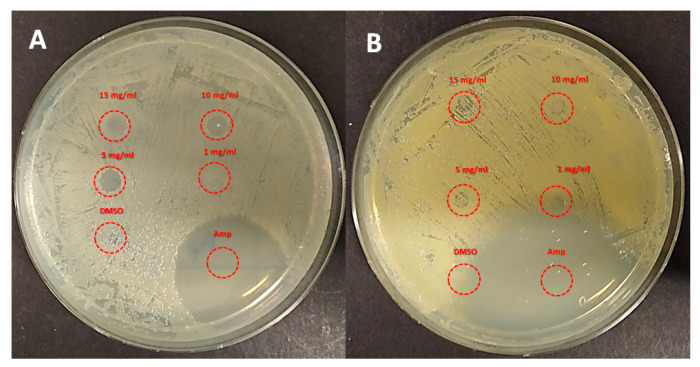
Antimicrobial effect of **CoPol1** at decreasing concentrations (15, 10, 5, and 1 mg/mL) on *E. coli* K12-MG1655 (**A**) and *S. aureus* ATCC 6538P (**B**), respectively. DMSO and ampicillin (x mg/mL) are included as controls.

As mentioned previously, the antimicrobial activity displayed by the conjugates could have an additional contribution from the polymers’ activity, beyond the expected effect of irradiated PSs (Figure S14). To verify the intrinsic antimicrobial activity of the copolymers, a different experimental setup was necessary. The former setup was useful to detect the antimicrobial activity induced by BODIPYs released from the copolymers upon irradiation. In the latter one, solution samples of **CoPol1** were administered, at increasing concentrations, to *E. coli* and *S. aureus* cells. As shown in Figure 4, a certain degree of inhibition of bacterial growth was observed in both strains, and especially in *E. coli*. 

## 3. Materials and Methods

All manipulations involving air-sensitive compounds were carried out under a nitrogen atmosphere using Schlenk or dry-box techniques. CuBr_2_, 2-2’-bipyridine (bpy), Sn (II) 2–ethylhexanoate (Sn(Oct)_2_), and bromoisobutyryl bromide (BiBB) were purchased from Sigma-Aldrich (Merck KGaA, Darmstadt, Germany) and used as received. Methyl methacrylate (MMA) and 2-(dimethylamino)ethyl methacrylate (DMAEMA) were purchased from Sigma-Aldrich and purified in a chromatographic column with basic alumina before use. Toluene and dichloromethane were dried over CaCl_2_ overnight before use.

The ^1^H NMR spectra were recorded on a Bruker AV400 (Bruker, Billerica, United States), operating at 400 MHz in the Fourier transform mode, and at 293 K. The samples (20 mg) were dissolved in 0.5 mL of CDCl_3_. Tetramethylsilane (TMS) was used as an internal chemical shift reference.

Differential scanning calorimetry analyses were performed on a NETZSCH STA 409 PC system (NETZSCH Geraetebau Gmbh, Selb, Germany). About 10 mg of sample was introduced into a 100 μL aluminum pan and heating/cooling cycles were registered at 20 K/min. In the first run, the sample was heated from 25 °C to 165 °C, followed by cooling to −60 °C. In the other two runs, the sample was heated from −60 °C to 165 °C, and cooled from 165 °C to −60 °C. The glass transition temperature (T*_g_*) of the samples was measured on the second heating run, where the inflection point is more easily detected.

Overnight cultures of *Escherichia coli* K MG1655 strain (F- lambda- ilvG- rfb-50 rph-1) and *Staphylococcus aureus* methicillin sensitive strain ATCC 6538P were grown in LB medium, at 37 °C, with agitation. LB agar plates were incubated at the same temperature. The recipe of LB (Lennox) medium is detailed below:

Tryptone: 10 g/L

Yeast extract: 5 g/L

NaCl: 5 g/L

(Agar: 15 g/L)

Sterilize by autoclaving

Overnight cultures of the tested strains were 10-fold sequentially diluted in 10 mL final volumes of sterile LB medium, according to the suitable experimental setup. Cell concentrations were evaluated with the viable count approach; briefly, 10 μL droplets of the suitable dilutions were spotted on LB agar plates and allowed to flow in a “top to bottom” fashion on inclined plates. After drying, the plates were incubated over night at 37 °C.

### 3.1. Synthesis of the BODIPYs

The BODIPYs 4,4-difluoro-2,6-diiodio-1,3,5,7-tetramethyl-8-(4-(4-bromobutoxy) phenyl)4-bora-3a,4a-diaza-s-indacene (**A**) and 4,4-difluoro-2,6-diiodo-1,3,5,7-tetramethyl-8-(4-[(8-bromooctoxy] phenyl)4-bora-3a,4a-diaza-s-indacene (**B**) were synthesized according to previously reported procedures [27,31].

### 3.2. General Procedure for the Copolymerization of MMA and DMAEMA by ARGET-ATRP

Under a nitrogen atmosphere, a 50 mL Schlenk flask was charged with anhydrous toluene (2 mL), CuBr_2_ (13.3 μmol, 37 μL, 36 mM in DMSO, 1 equiv.), 2-2’-bipyridine (4.8 equiv.), Sn(Oct)_2_ (100 equiv.), and bromoisobutiryl bromide (BiBB, 12.5 equiv.). The flask was thermostated at 60 °C, then the desired amounts of MMA and DMAEMA were introduced. The reaction was quenched after 18 h by adding 30 mL of *n*-hexane. The copolymer was recovered by filtration, dissolved in acetone, reprecipitated from *n*-hexane (30 mL), recovered by filtration, and finally dried at 60 °C under reduced pressure.

^1^H NMR (CDCl_3_) δ: 0.86–1.23 (3H, -C*H*_3_); 1.80 (2H, chain -C*H*_2_); 2.30 (6H, -N(C*H*_3_)_2_); 2.59 (2H, -OC*H*_2_-C*H*_2_N); 3.57 (2H, -OC*H*_3_); 4.07 (2H, OC*H*_2_-C*H*_2_N).

The copolymers chemical compositions, as mol% of DMAEMA, were evaluated by ^1^H NMR spectroscopy from integrals of signals at 3.57 and 4.07 ppm according to the following equations:

*χ_DMAEMA_* = {*I*_4.07ppm_/[(*I*_4.07ppm_/2) + (*I*_3.57ppm_/3)]} 100% *χ*_MMA_ = 1 − *χ_DMAEMA_*

### 3.3. Binding of BODIPYs A and B to P(MMA-ran-DMAEMA) Copolymers

The binding of the A/B BODIPYs onto the copolymers was performed as follows:

A 50 mL round flask, equipped with a refrigerator, was charged with acetone, P(MMA-*ran*-DMAEMA) copolymer, and BODIPY in a ratio of 0.5 mL/50 mg polymer/1 mg BODIPY. The flask was thermostated at 50 °C and the reaction was carried out for 25 h under stirring. The reaction was quenched by the addition of 20 mL *n*-hexane. The product was recovered by filtration and washed several times with diethyl ether until all unreacted BODIPY was removed, as proved by TLC (CH_2_Cl_2_ as eluent). Once purified, the product was solubilized in CH_2_Cl_2_, reprecipitated in *n*-hexane, and dried in vacuum at 60 °C overnight.

The purity of the products was then verified as follows: a 10 mg sample was dissolved in the minimum amount of methanol and eluted through a Pasteur pipette, packed with Sephadex^®^ LH-20 as the stationary phase, by using methanol as the eluent. The eluted product was then collected inside a 96-multiwell plate, picking up 6 drops of eluate per well. The amounts of BODIPY covalently bound to the copolymers were evaluated via UV analyses of the various collected fractions on a TECAN instrument, by measuring the absorbance of each well at λ = 535 nm.

### 3.4. Preparation of Discs for Microbiological Assays

The solution of copolymers, or labelled copolymers, in methanol, was added dropwise by sequential addition of 10 µL volumes onto filter paper (pore sizes 8–12 μm) discs with diameters of 10 mm, using a spin coater device (SCI-30 model—Keramik und Labortechnik Markkleeberg), applying a centrifuge field. Once the solvent was evaporated, thin layers were generated. The amount of dissolved polymers was calculated to have a normalized concentration of the corresponding photosensitizers.

### 3.5. Antimicrobial Assays

*Escherichia coli* K12-MG1655 and *Staphylococcus aureus* methicillin sensitive ATCC 6538P were chosen as model strains. Isolated bacterial colonies were inoculated in 20 mL of LB rich medium in Erlenmeyer flasks and grown overnight at 37 °C under mild agitation. Bacterial samples of 100 μL at ~10^7^ CFU/mL were inoculated on LB agar, and the coated paper discs were placed on the solid medium surface. In addition, control discs were prepared with copolymer or methanol. The bacteria and discs were incubated in the dark for 2 h at 37 °C, and then irradiated under LED (530 nm, 3.036 × 10^−3^ W/cm^2^) for 7 h at room temperature, to reach a fluence rate of 76.5 J/cm^2^. A dark control was included for each experiment. After incubation at 37 °C, the antimicrobial effect was evaluated by measuring the diameters of the inhibitory growth halo around the filter paper discs. The experiments were performed at least three times with independent cultures.

To verify the photoactivity of BODIPYs A and B, *E. coli* and *S. aureus* cells at 10^7^ CFU/mL were inoculated on LB agar, and droplets of 10 μL of solvent alone or BODIPYs at decreasing concentrations (100, 50, 25, 12.5, and 6.25 μM) were added. After 2 h of dark incubation, cells were irradiated (76.5 J/cm^2^). After overnight incubation at 37 °C, the inhibitory effect on bacterial growth was checked. The same experimental setup, under dark conditions, was applied, to test the antimicrobial effect of decreasing concentrations of **CoPol1** (15, 10, 5, and 1 mg/mL). Samples of DMSO and ampicillin 100 μM were included as controls.

## 4. Conclusions

The antimicrobial photodynamic activity of six P(MMA-*ran*-DMAEMA) copolymers bound to two different BODIPYs was evaluated. They were shown to kill bacteria within minutes, by triggering the PS with light, and to display a *permanent* bactericidal character thanks to the quaternary ammonium groups and protonated amino groups of the copolymers, the latter owing to their unconjugated DMAEMA units. Under the tested conditions, *S. aureus*, that is the etiological agent of many skin infections, seems the ideal target of the proposed light-based treatment. Among the tested BODIPY-conjugated copolymers, **CoPol2-B** proved to have the highest efficiency in photo-inactivating *S. aureus*, possibly because of the specific morphology of the coating, allowing higher exposure of the PS. In order to corroborate this assumption, morphology studies are still ongoing in our laboratories.

Remarkably, these experiments demonstrated the potential use of such systems as wound dressing bandages, that combine the instantaneous photodynamic action of the BODIPYs with the long-term bactericidal action of the copolymer. In order to verify the applicability of these antimicrobial polymers on other Gram-positive bacteria, further investigations are still ongoing in our laboratories.

## Figures and Tables

**Table 1:** Synthesis of P(MMA-*ran*-DMAEMA) copolymers by (ARGET-ATRP).

Entry	Sample ID	χ_DMAEMA_^feed^	χ_DMAEMA_ ^a^	*T*_g_ ^b^ (°C)
1	**CoPol1**	0.50	0.51	72
2	**CoPol2**	0.33	0.43	65
3	**CoPol3**	0.25	0.29	90

^a^ Determined by ^1^H NMR spectroscopy on the resulting polymer. ^b^ Determined by DSC.

**Table 2:** BODIPY content in P(MMA-*ran*-DMAEMA) copolymers.

Entry	Sample ID	BODIPY	χ_DMAEMA_ (%)	%_BODIPY_ (mg_BODIPY_/mg_copolymer_)	mol_BODIPY_/g_copolymer_	*T*_g_ (°C)
1	**CoPol1-A**	**A**	51	1.02	1.40 × 10^−5^	75
2	**CoPol1-B**	**B**		1.25	1.59 × 10^−5^	80
3	**CoPol2-A**	**A**	43	0.70	9.57 × 10^−6^	83
4	**CoPol2-B**	**B**		0.75	9.40 × 10^−6^	84
5	**CoPol3-A**	**A**	29	1.23	1.58 × 10^−5^	100
6	**CoPol3-B**	**B**		0.51	6.34 × 10^−6^	93

**Table 3:** Antimicrobial activity in *E. coli* K12-MG1655. Diameters (mm) of inhibitory haloes are reported as mean ± sd of at least three independent assays.

Entry	Sample ID	M_CoPol_ (μg)	n_BODIPY_ (μmol)	MG1655 Zone of Inhibition (mm)			Average (mm)
				Replicates			
				I	II	III	
1	**CoPol1-A**	712	0.01	15.0	13.4	18.9	15.77 ± 2.83
2	**CoPol1-B**	630	0.01	11.9	13.1	15.3	13.43 ± 1.72
3	**CoPol2-A**	1040	0.01	16.5	15.8	15.4	15.90 ± 0.56
4	**CoPol2-B**	1060	0.01	16.0	16.8	16.8	16.53 ± 0.46
5	**CoPol3-A**	630	0.01	15.6	14.0	11.7	13.77 ± 1.96
6	**CoPol3-B**	1570	0.01	13.2	15.7	13.7	14.20 ± 1.32

**Table 4:** Antimicrobial activity in *S. aureus* ATCC 6538P. Diameters (mm) of inhibitory haloes are reported as mean ± sd of at least three independent assays.

Entry	Sample ID	M_CoPol_ (μg)	n_BODIPY_ (μmol)	MSSA Zone of Inhibition (mm)			Average (mm)
				Replicates			
				I	II	III	
1	**CoPol1-A**	712	0.01	25.7	19.5	30.0	25.07 ± 5.28
2	**CoPol1-B**	630	0.01	21.8	26.8	26.4	25.00 ± 2.78
3	**CoPol2-A**	1040	0.01	32.9	22.9	30.3	28.60 ± 5.14
4	**CoPol2-B**	1060	0.01	40.2	26.7	31.6	32.83 ± 6.83
5	**CoPol3-A**	630	0.01	27.1	23.4	19.0	23.17 ± 4.06
6	**CoPol3-B**	1570	0.01	24.5	23.4	22.0	23.30 ± 1.25

## Data Availability

Not applicable.

## Supplementary Materials

The following supporting information can be downloaded at: https://www.mdpi.com/article/10.3390/ijms24054340/s1.
