# Supplementary material for: Inherently Antimicrobial P(MMA-ran-DMAEMA) Copolymers Sensitive to Photodynamic Therapy: A Double Bactericidal Effect for Active Wound Dressing
Source: Int J Mol Sci. 2023 Feb 22;24(5):4340. doi: 10.3390/ijms24054340 (PMC10001570; doi:10.3390/ijms24054340)
Supplement: Supplementary file 1 [file ijms-24-04340-s001.zip › ijms-2113646-supplementary.pdf]

Supplementary material for

Inherently Antimicrobial P(MMA-*ran*-DMAEMA) Copolymers Sensitive to Photodynamic Therapy: A Double Bactericidal Effect for Active Wound Dressing

Orlando Santoro,[1] Miryam Chiara Malacarne,[1] Francesco Sarcone,[1] Luca Scapinello,[1] Stefania Pragliola,[2] Enrico Caruso,[1] Viviana Teresa Orlandi,[1] Lorella, Izzo[1]*

[1] Dipartimento di Biotecnologie e Scienze della Vita, via J.H. Dunant, 3 – 21100 Varese

[2] Dipartimento di Chimica e Biologia, via Giovanni Paolo II, 128 – 85085 Fisciano (SA)

Contents

General Information ... S3
^{1}H NMR Spectra of the copolymers ... S3
DSC curves of the copolymers ... S7
Microbiological Assay ... S12

General Information

All manipulations involving air-sensitive compounds were carried out under nitrogen atmosphere using Schlenk or dry-box techniques.

$CuBr_2$, 2-2'-bipyridine (bpy), Tin (II) 2–ethylhexanoate ($Sn(Oct)_2$), and bromoisobutyryl bromide (BiBB) were purchased from Sigma Aldrich and used as received. Methyl methacrylate (MMA) and 2-(dimethylamino)ethyl methacrylate (DMAEMA) were purchased from Sigma Aldrich and purified in a chromatographic column with basic alumina before use. Toluene and dichloromethane were dried over $CaCl_2$ overnight before use.

^{1}H NMR spectra were recorded on a Bruker AV400 operating at 400 MHz in the Fourier Transform mode and at 293 K. The samples (20 mg) were dissolved in 0.5 mL of $CDCl_3$. Tetramethylsilane (TMS) was used as an internal chemical shift reference.

^{1}H NMR Spectra of the copolymers

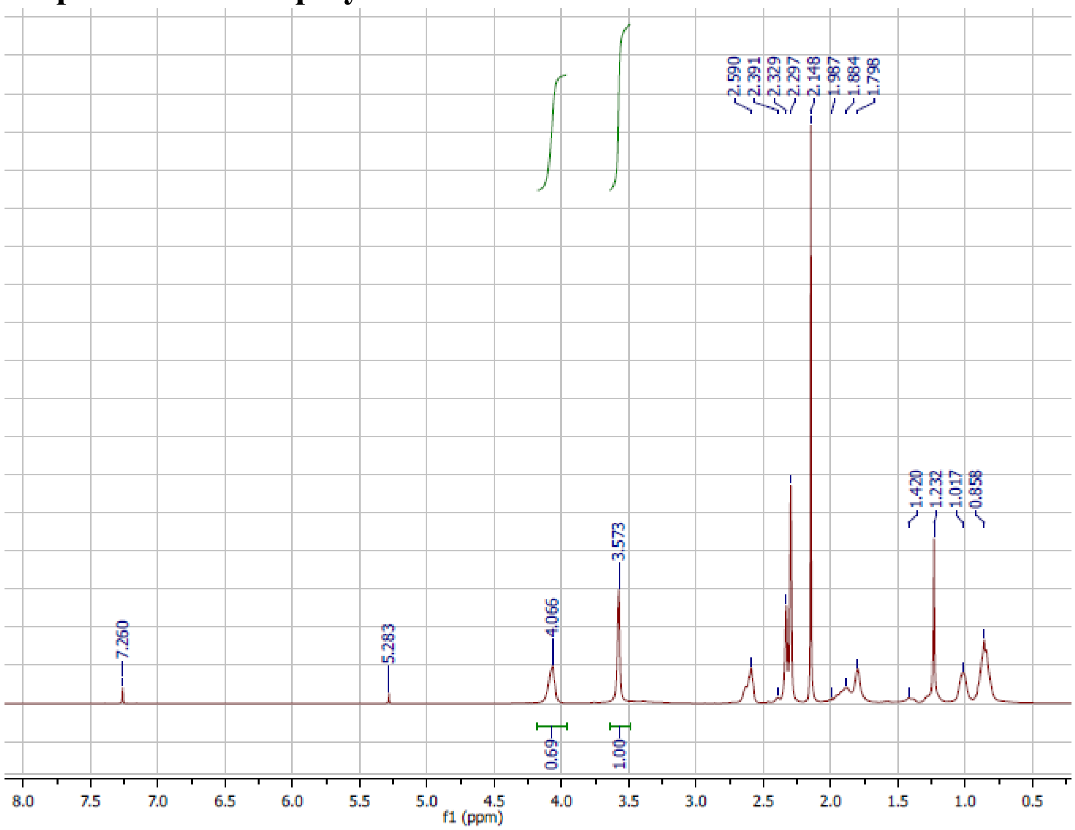

Figure S1. ^{1}H NMR spectrum ($CDCl_3$, 400 MHz, 25 °C) of **CoPol1** (Table 1, entry 1).

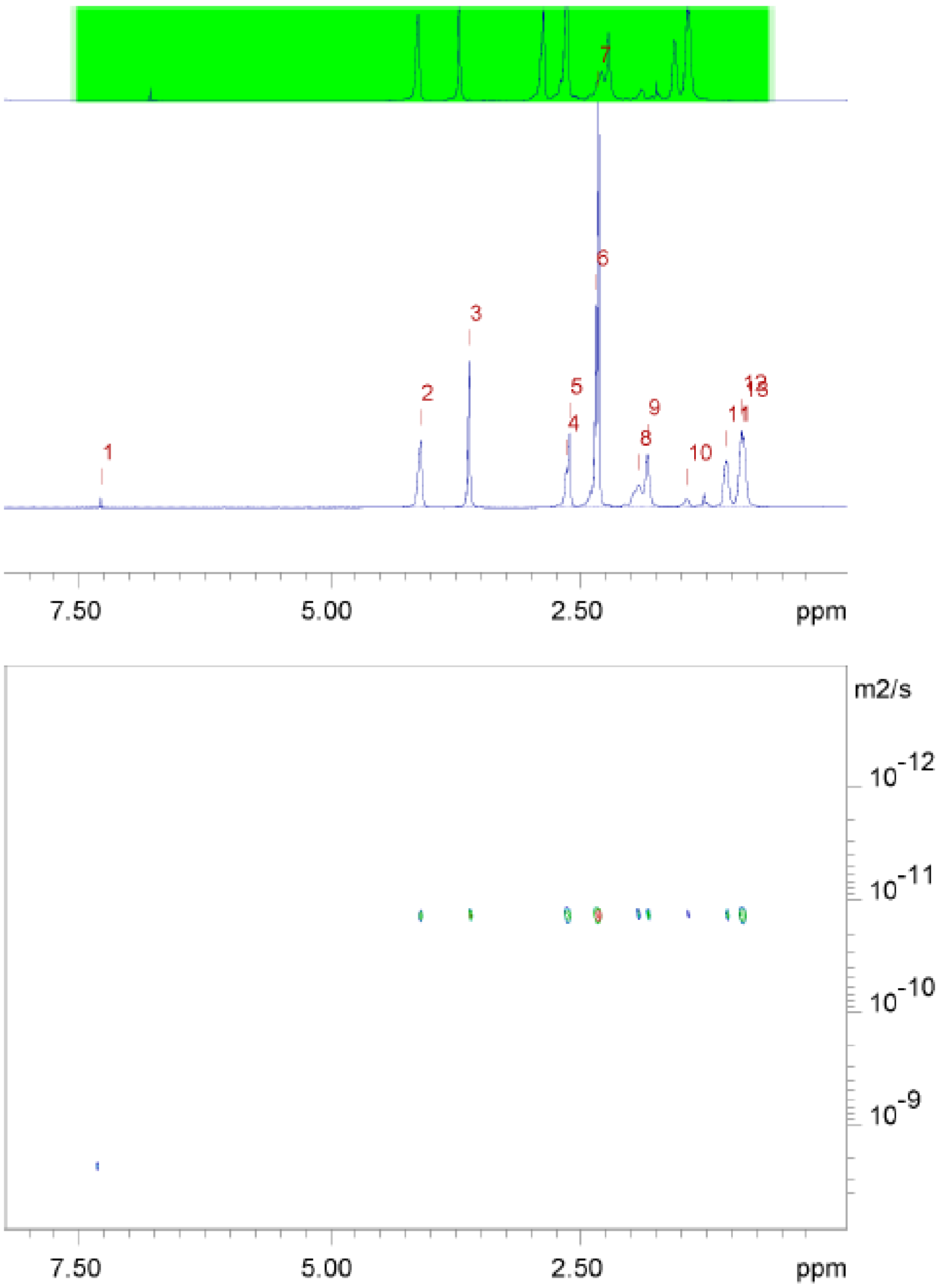

Figure S2. ^{1}H DOSY NMR spectrum ($CDCl_3$, 400 MHz, 25 °C) of **CoPol1** (Table 1, entry 1).

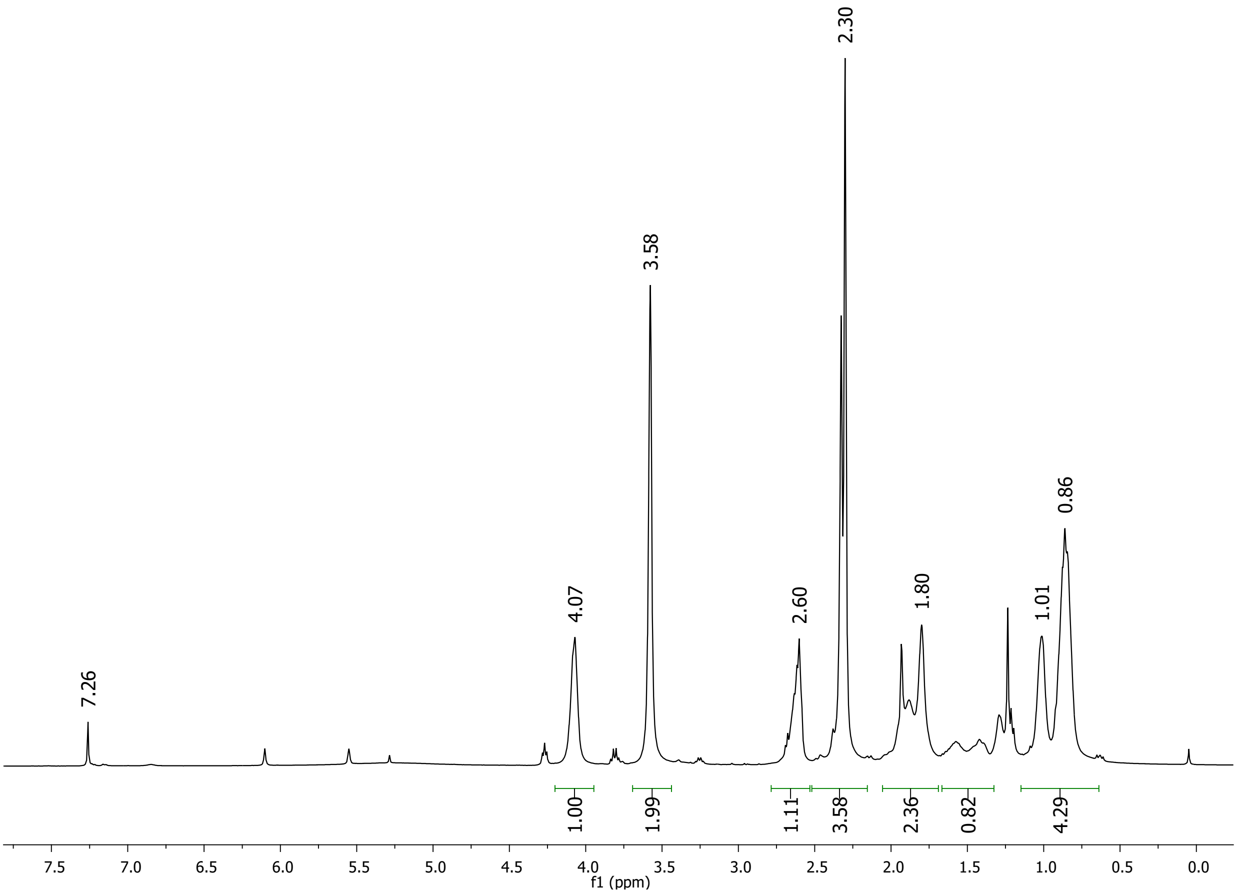

Figure S3. ^{1}H NMR spectrum ($CDCl_3$, 400 MHz, 25 °C) of **CoPol2** (Table 1, entry 2).

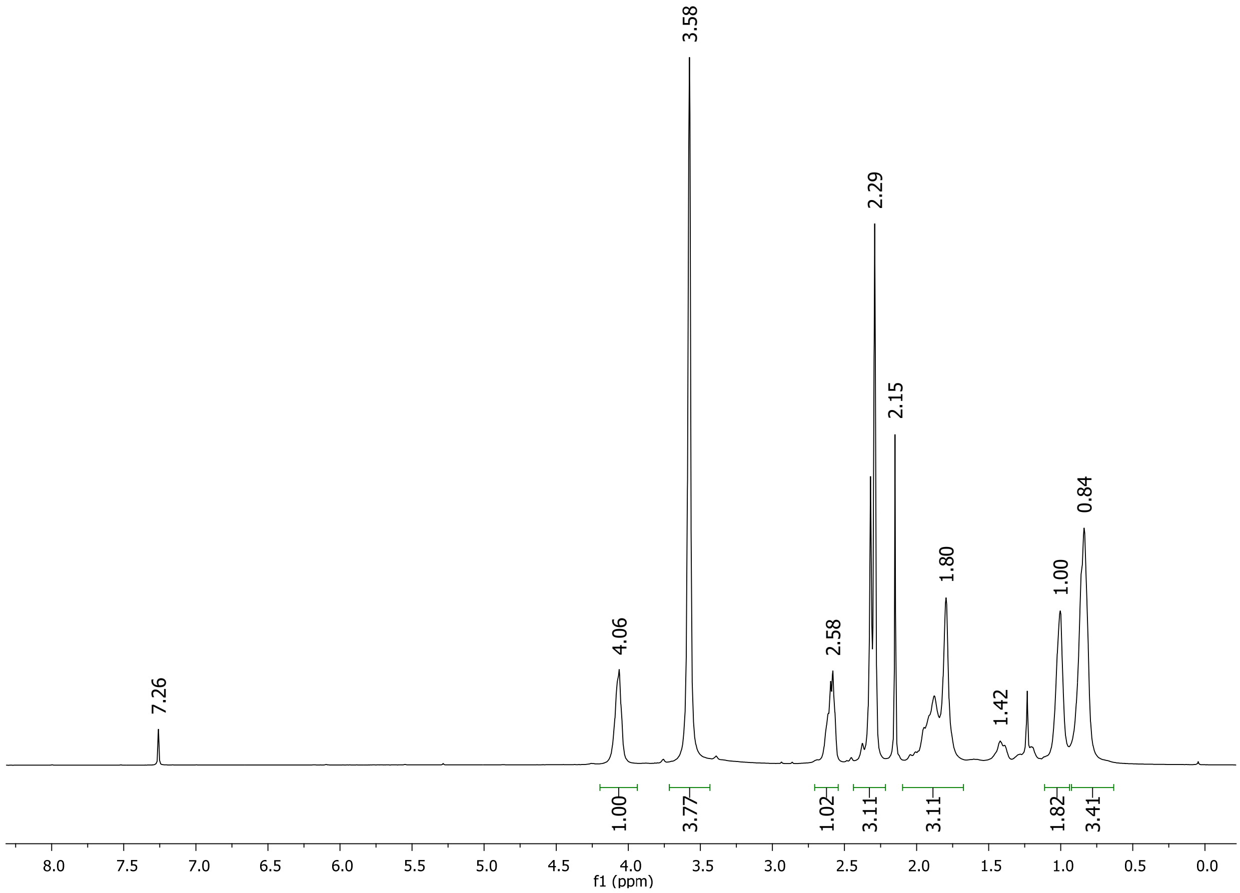

Figure S4. ^{1}H NMR spectrum ($CDCl_3$, 400 MHz, 25 °C) of **CoPol3** (Table 1, entry 3).

DSC curves of the copolymers

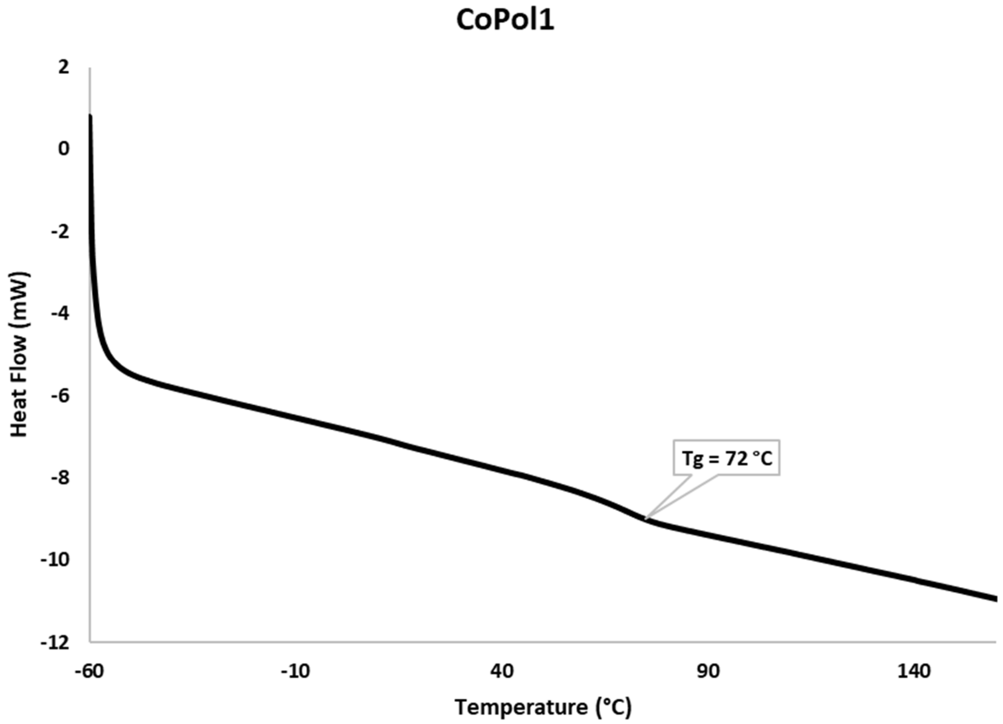

Figure S5. DSC curve for **CoPol1** (Table 1, entry 1, second heating step).

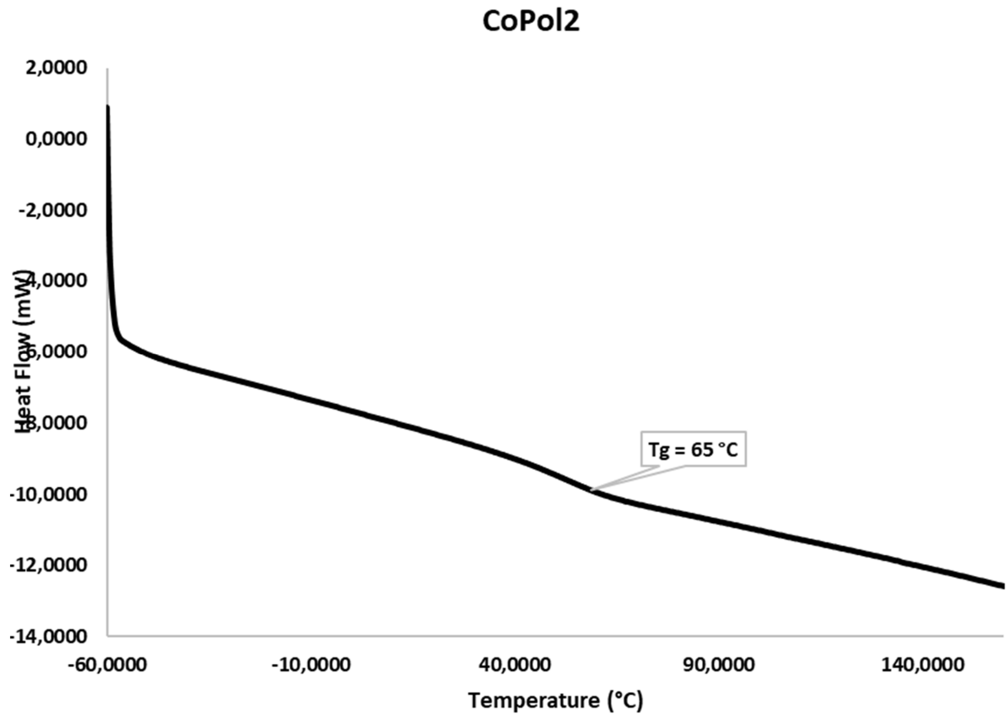

Figure S6. DSC curve for **CoPol2** (Table 1, entry 2, second heating step).

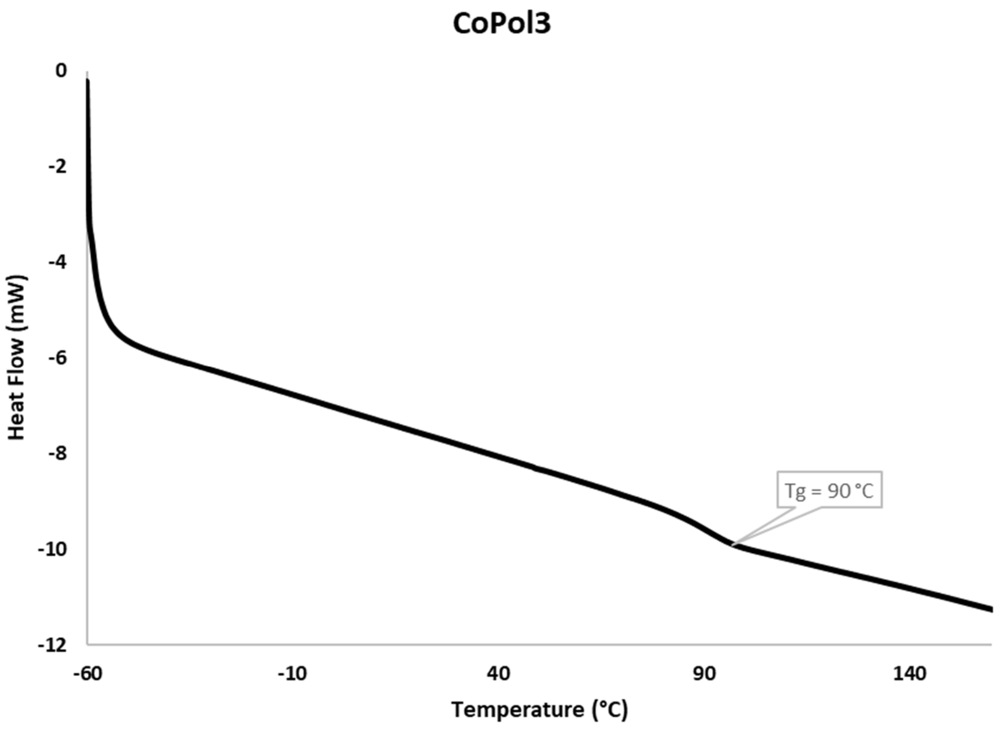

Figure S7. DSC curve for **CoPol3** (Table 1, entry 3, second heating step).

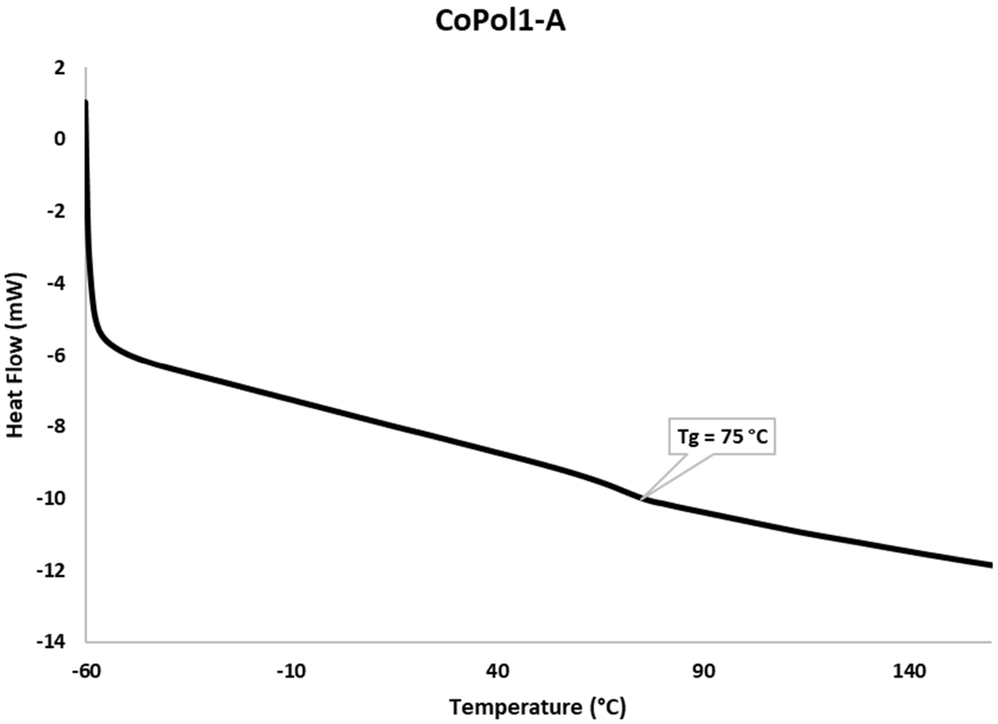

Figure S8. DSC curve for **CoPol1-A** (Table 2, entry 1, second heating step).

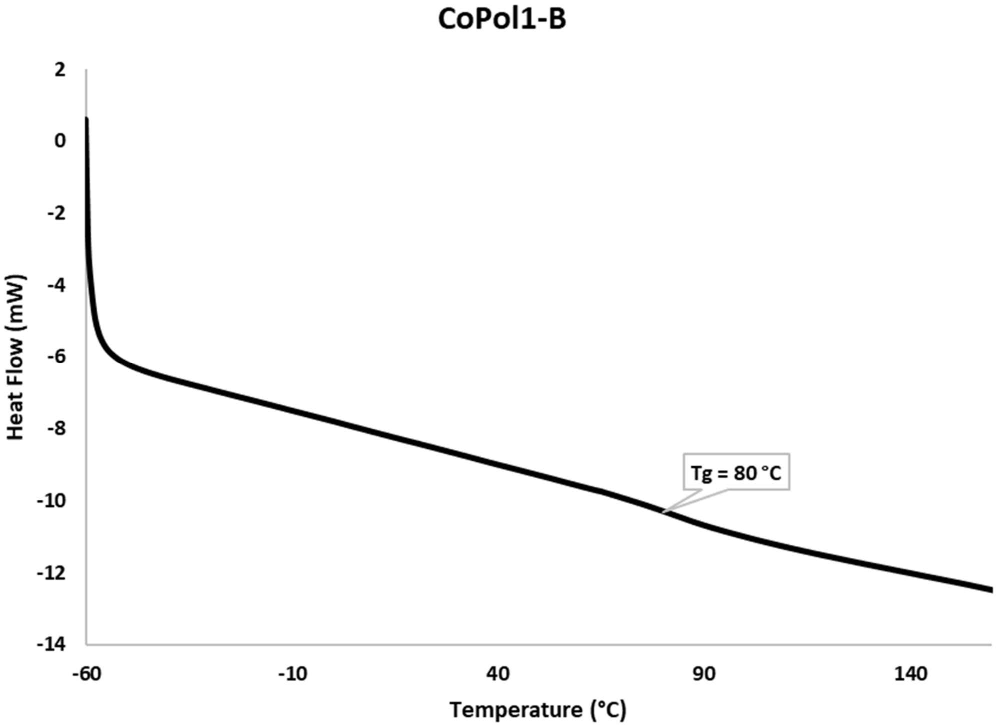

Figure S9. DSC curve for **CoPol1-B** (Table 2, entry 2, second heating step).

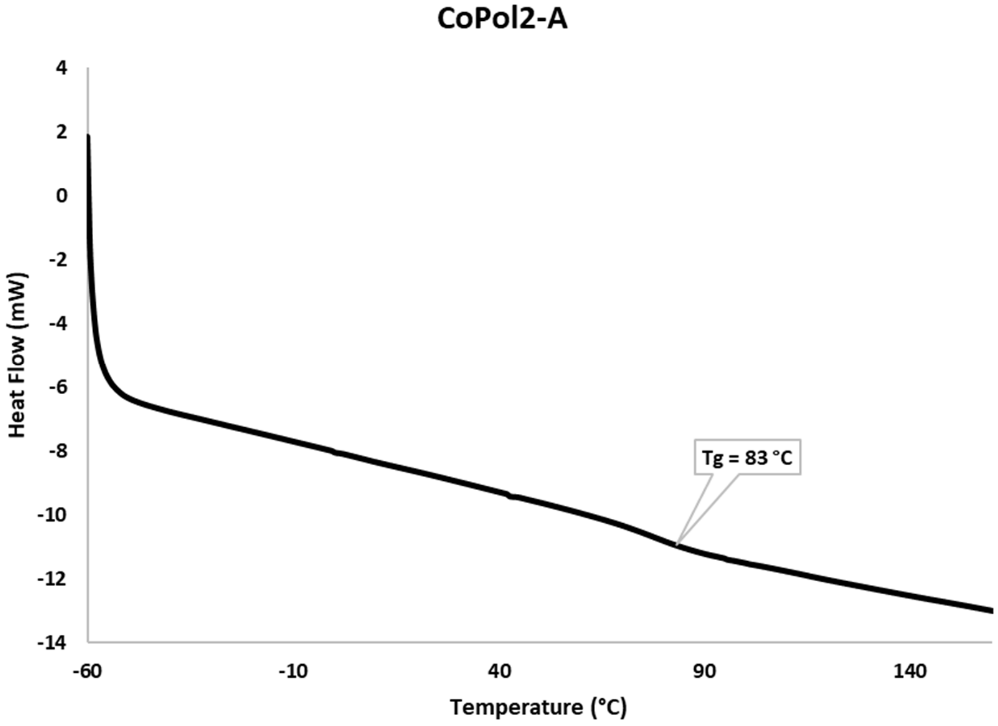

Figure S10. DSC curve for **CoPol2-A** (Table 2, entry 3, second heating step).

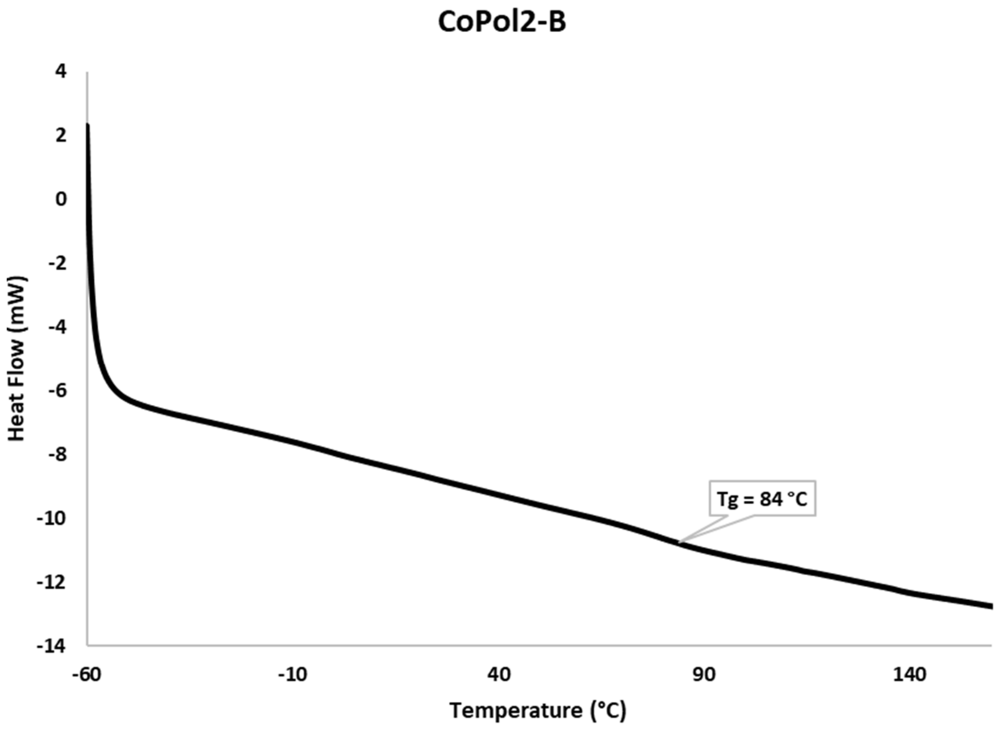

Figure S11. DSC curve for **CoPol2-B** (Table 2, entry 4, second heating step).

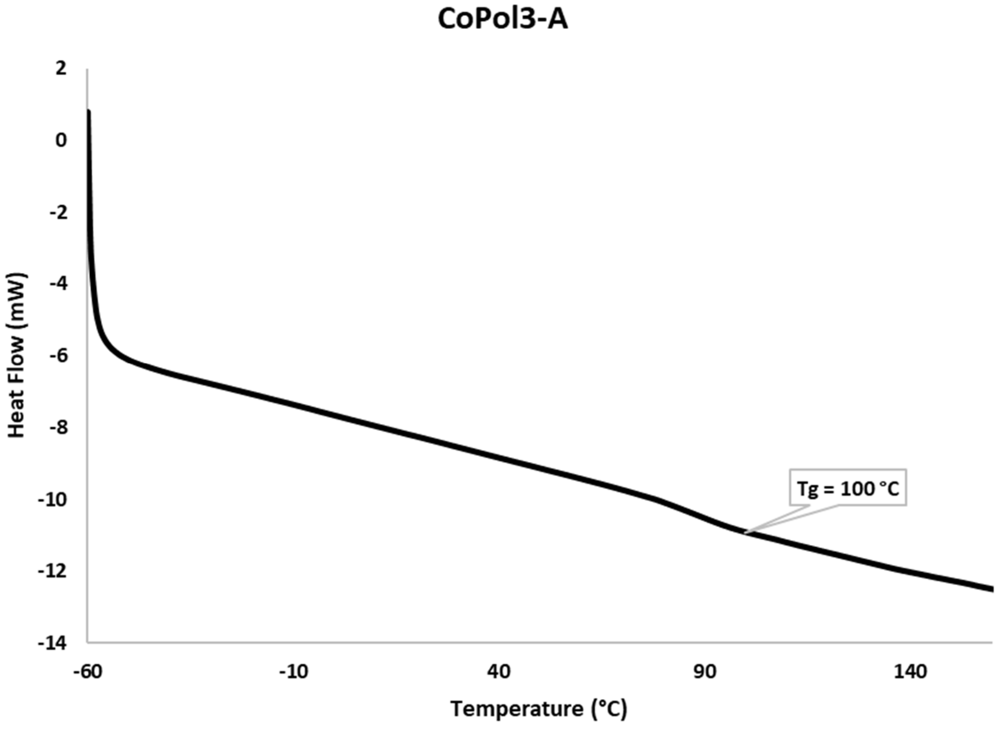

Figure S12. DSC curve for **CoPol3-A** (Table 2, entry 5, second heating step).

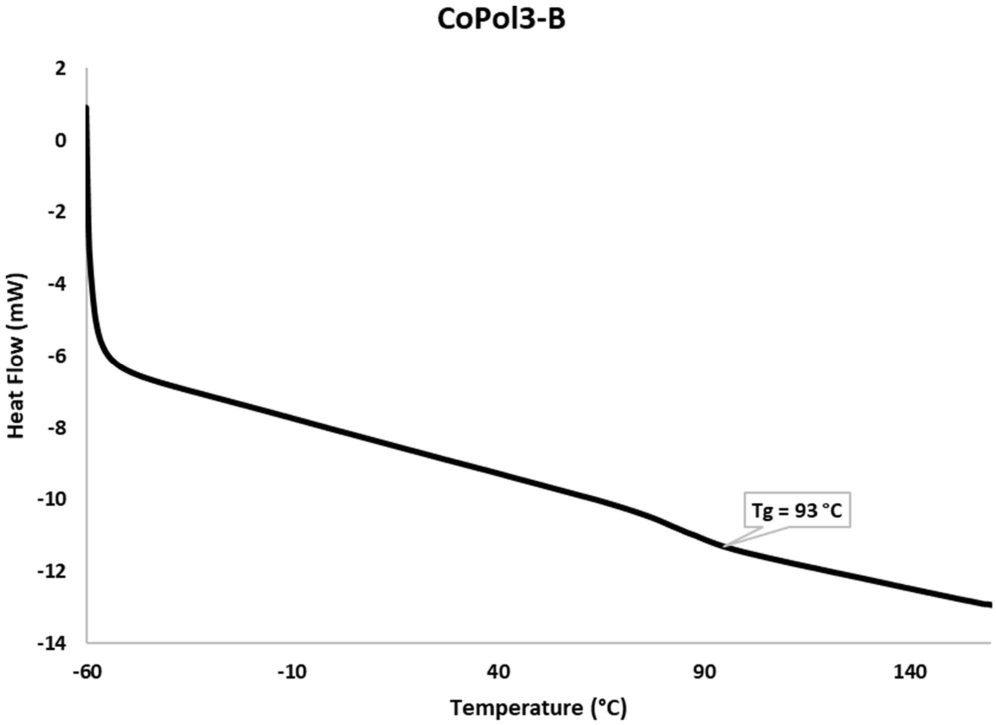

Figure S13. DSC curve for **CoPol3-B** (Table 2, entry 5, second heating step).

Microbiological Assay

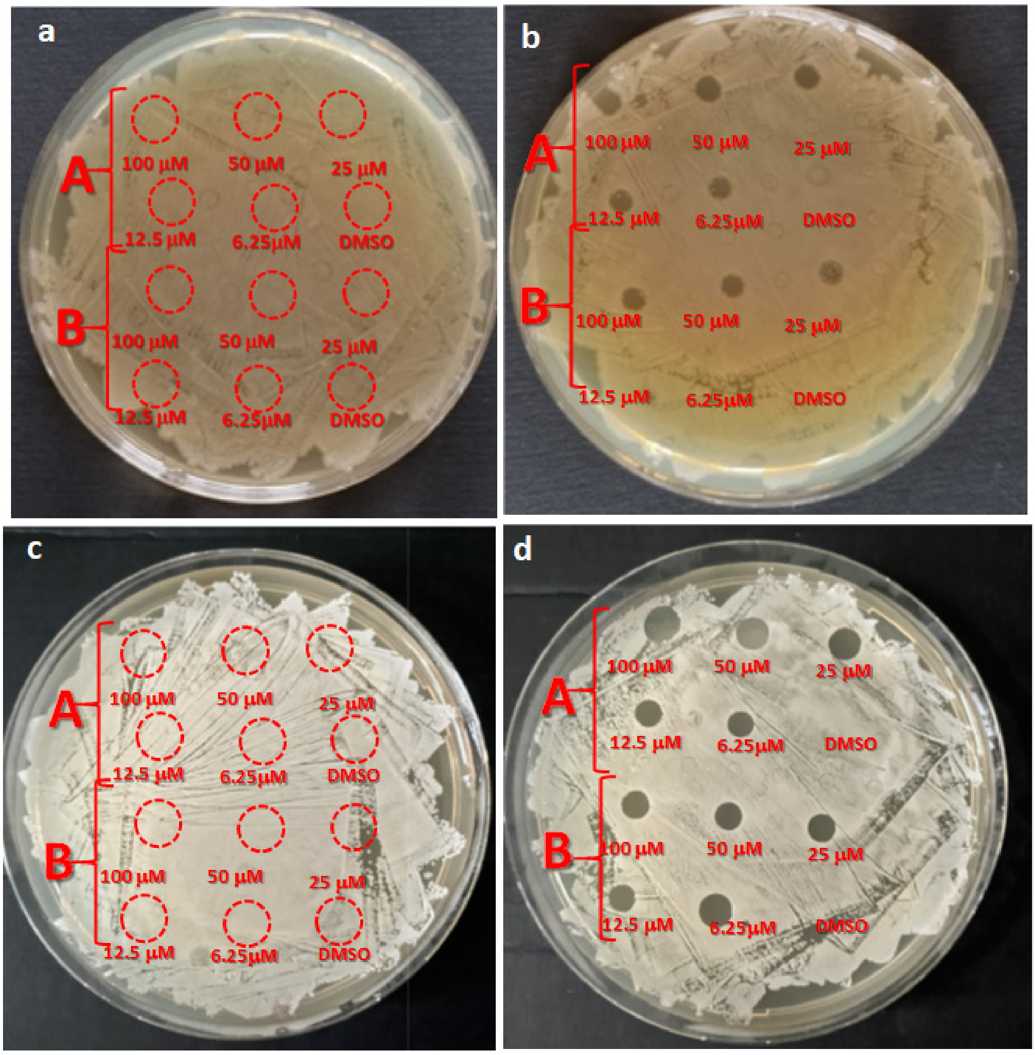

Figure S14: Effect of BODIPYs A and B on *E. coli K12-MG1655* (a,b) and *S. aureus ATCC 6538P* (c,d). BODIPYs A and B were administered at decreasing concentrations (100, 50, 25, 12.5 and 6.25 mM) to cells. After 2 hours of dark incubation, cells were irradiated (76.5 J/cm^2) or dark incubated. After incubation at 37°C the inhibitory effect is clearly observable.
